# Ocular Fluid As a Replacement for Serum in Cell Cryopreservation Media

**DOI:** 10.1371/journal.pone.0131291

**Published:** 2015-07-02

**Authors:** Vivek Phani Varma, Lalitha Devi, Naresh Kumar Venna, Ch Lakshmi N. Murthy, Mohammed M. Idris, Sandeep Goel

**Affiliations:** Centre for Cellular and Molecular Biology, Council for Scientific and Industrial Research, Hyderabad, India; French Blood Institute, FRANCE

## Abstract

Cryostorage is of immense interest in biomedical research, especially for stem cell-based therapies and fertility preservation. Several protocols have been developed for efficient cryopreservation of cells and tissues, and a combination of dimethyl sulfoxide (DMSO) and fetal bovine serum (FBS) is commonly used. However, there is a need for an alternative to FBS because of ethical reasons, high cost, and risk of contamination with blood-borne diseases. The objective of the present study was to examine the possibility of using buffalo (*Bubalus bubalis*) ocular fluid (BuOF) to replace FBS in cryomedia. Frozen–thawed cells, which were cryopreserved in a cryomedia with BuOF, were assessed for viability, early and late apoptosis, and proliferation. Three cell lines (CHO, HEK, and C18-4), mouse embryonic stem (mES) cells, and primary cells, such as mouse embryonic fibroblast (MEF) cells, human peripheral blood mononuclear cells (hPBMCs), and mouse bone marrow cells (mBMCs), were cryopreserved in cryomedia containing 10% DMSO (D10) with 20% FBS (D10S20) or D10 with 20% BuOF (D10O20). For all three cell lines and mES cells cryopreserved in either D10S20 or D10O20, thawed cells showed no difference in cell viability or cell recovery. Western blot analysis of frozen–thawed-cultured cells revealed that the expression of Annexin V and proliferating cell nuclear antigen (PCNA) proteins, and the ratio of BAX/BCL2 proteins were similar in all three cell lines, mES cells, and hPBMCs cryopreserved in D10S20 and D10O20. However, initial cell viability, cell recovery after culture, and PCNA expression were significantly lower in MEF cells, and the BAX/BCL2 protein ratio was elevated in mBMCs cryopreserved in D10O20. Biochemical and proteomic analysis of BuOF showed the presence of several components that may have roles in imparting the cryoprotective property of BuOF. These results encourage further research to develop an efficient serum-free cryomedia for several cell types using BuOF.

## Introduction

Cryopreservation is a technique that enables preservation of structure and function of cells and tissues by preventing or minimizing damage. Cryopreserved cells and tissues can be stored for prolonged periods in limited space at ultra-low temperatures and require less maintenance [1]. Stem cells are used in transplantation and cell-based therapies, which makes these cells a promising tool for regenerative and biomedical research. However, stem cells have a limited lifespan [2], and with increase in the number of passages, their proliferative capacity and differentiation decrease [3, 4]. Embryonic stem (ES) cells are derived from the inner cell mass of blastocyst-stage embryoswith the potential to self-renew and differentiate into different cell lineages [5]. Mouse ES cells are used in various research applications including gene targeting, which helpsgeneratetarget mutant mouse models for functional genomic studies and thereputic applications [6]. Spermatogonial stem cells (SSCs)isolated from donor mice repopulated and produced mature spermatozoa in recipient mouse testes [7]. Maintenance of proliferative and differentiation capacity of ES cells and SSCs when subjected to cryopreservation could extend their applications for regenerative therapy and restoration of fertility in cancer patients who are rendered infertile because of the gonadotoxic effects of chemotherapy and radiotherapy [6].

Several established cell lines of different origins are routinely used for drug assays and gene expression analyses [8]. Various primary cells are also routinely used in biological research. Mouse embryonic fibroblast (MEF) cells are used as feeder cells to maintain ES cells and induced pluripotent stem (iPS) cells in the undifferentiated state [9, 10]. Peripheral blood mononuclear cells (PBMCs) are commonly used for prospective phenotypic and functional analyses in a wide range of infectious disease and clinical vaccine studies [11]. PBMCs are used to develop effective human immunodeficiency virus1 vaccines and infer effector function and cellular or humoral immune responses [12]. Mouse bone marrow cells (mBMCs) are used to study angiogenesis inhibitor drugs in cancer treatment [13, 14]. These cells have potential applications in hematopoietic stem cell transplantation research [15] and regenerative medicine [16]. Each cell type differs in structure and cell membrane composition because they respond differently to cryopreservation. Culture age at the time of freezing and cultivation procedures may also impact freezing success [17]. Current widespread increase in the application of cell culture methods to various areas of biology and medicine emphasizes the need for contamination-free and efficient cell cryopreservation.

During cryopreservation, cellular injury primarily occurs when water, which is an important cell constituent, freezes at ultralow temperatures. Cellular injury can be minimized by adding cryoprotective agents (CPAs). Dimethyl sulfoxide (DMSO) is a widely used permeable CPAs in cell and tissue cryopreservation because of its low toxicity [18]. Fetal bovine serum (FBS) is a commonly used non-permeable CPA that is combined with DMSO for several cell lines and tissues because it supports better cell recovery. However, there is a need to find a replacement for FBS as a cryopreservative because it is expensive. Furthermore, the methods for harvesting blood are inhumane because FBS is harvested from bovine fetuses taken from pregnant cows during slaughter by means of cardiac puncture without any anesthesia [19, 20]. Use of animal serum is also associated with a risk of contamination with viruses [21] and prions as well as possible disease transmission [22], some of which are impossible to remove from serum. Some of these infectious agents, such as bacteria and viruses, are even capable of surviving at low temperatures that are routinely used for cell stock storage (-160°C) [23, 24]. Although FBS is indispensable in biomedical research, FBS-free cryomedia would benefit researchers by conforming to good laboratory practices.

Several alternatives to serum in cryomedia have been considered. Sericin is a protein hydrolysate that is rich in serine and obtained from raw silk during the degumming process. Expression of sericin in *Escherichia coli* has been shown to prevent freezing stress and promote cell viability [25]. A medium that contained sericin-cryopreserved Chinese-hamster ovary (CHO) and P3UI myeloma cells was as efficient as the conventional FBS-containing medium and superior to several commercial serum-free freezing media[26]. Similarly, replacement of FBS with bovine serum albumin (BSA) in cryomedia showed no significant difference in cell recovery and viability of peripheral blood mononuclear cells [27]. However, the extremely high costs of sericin and BSA limit their use, especially in developing countries.

Bovine ocular fluid (BOF) alone and in combination with sheep plasma and human serum albumin has been evaluated as a serum replacement for different cells [28]. BOF has several active components that promote cell growth, such as vascular endothelial growth factor [29], the 21-KDa acidic-Ca-binding protein [30], insulin like growth factor, hypoxanthine, and fibronectin [31]; however, it does not support cell growth on its own. In addition, ocular fluid has several proteins, including albumin [32], which may act as non-permeable CPAs. Collection of buffalo ocular fluid (BuOF) is feasible in India because there is no religious taboo regarding buffalo slaughter, and buffalo eyes are available in abundance as a slaughter house by-product. The price of BuOF is approximately 7–8-fold lower than that of FBS (in Indian scenario), and aseptic collection of BuOF is possible because eyes are enclosed organs. However, the effect of BuOF on cell cryopreservation has not been evaluated to date.

The aim of the present study was to evaluate if BuOF can replace FBS for cell cryopreservation. We also evaluated the composition of BuOF to identify the component(s) that may play a key role in imparting cryoprotective ability.

## Materials and Methods

### Collection of BuOF

All animal procedures were approved by the Institute Animal Ethics Committee (IAEC) of Centre for Cellular and Molecular Biology (CCMB), Hyderabad, India. Intact eyeballs from healthy Murrah male calves (n = 12; age, 6–8 months) were collected from the Municipal Slaughter House, Hyderabad, India and transported in phosphate buffered saline (PBS; Invitrogen) on ice. After arrival to the laboratory within 1 h of slaughter, ocular muscles and the optic nerve were trimmed from the eyeballs. After rinsing with ice cold 50% alcohol and PBS several times, the cornea of the eyeball was carefully punctured with a 22-gauge needle, and aqueous humor was collected. The posterior chamber of the eyeball was then cut open using a sterile surgical blade, and the vitreous humor was collected using a 10-ml syringe. Collected aqueous and vitreous humor were pooled from all animals in a given trial (n = 3), centrifuged at 775 × *g* (15 min at 4°C), and then filtered through 0.45-μm and 0.22-μm filters. The sterile BuOF was aliquoted into cryovials (Nunc) and stored at -30°C until use.

### Biochemical analysis of BuOF and FBS

Differences in biochemical composition of BuOF and FBS were determined by a medical diagnostic agency (Vijaya Diagnostics; www.vijayadiagnostic.com). A total of three samples each for FBS (Gibco; origin, United States; lot numbers; 494515, 816712, and 835987 respectively) and pooled BuOF were used for analysis. The mean value for each analyzed biochemical and the methods used for analysis are listed in Table 1.

**Table 1:** Comparative biochemical analysis of BuOF and FBS.

	Parameters (mg/dl)	Method used for analysis	BuOF	FBS
1	Total Protein	Biuret	0.5 ± 0.03	3.25 ± 0.23
2	Albumin	Bromocresol green (BCG)	0.2 ± 0.09	1.85 ± 0.32
3	Globulin	Biuret and BCG	0.3 ± 0.01	1.4 ± 0.2
4	Lipoprotein A	Particle enhanced Immunoturbidimetry	1.8 ± 0.1	2.5 ± 0.2
5	Triglycerides	Glycerol-3-phosphate oxidase- phenol aminophenazone (GPO-PAP)colorimetry	13 ± 0.9	61 ± 1.4
6	Total Cholesterol	(Cholesterol oxidase- peroxidase enzyme) isotope dilution (CHO-POD) IDMS mass spectroscopy	22.5 ± 1	27.5 ± 1.02
7	LDL Cholesterol	Enzymatic immunoinhibition	14 ± 0.9	8 ± 0.8
8	HDL Cholesterol	(Total cholesterol)–(HDL + VLDL cholesterol)	6 ± 0.7	8 ± 0.6
9	VLDL Cholesterol	(TG /5)	2.6 ± 0.5	12.5 ± 0.8
10	Glucose	Hexokinase	56.5 ± 2.3	77 ± 4.1
11	Ascorbic acid	Spectrophotometry	7.27 ± 0.7	1.13 ± 0.2

Values are represent in mean ± SEM.

### Cell lines, derivation of primary cells, and cell culture

Derivation of cells from mice was approved by IAEC (permit numbers IAEC 52/2014 and 4/2015). For isolation of human peripheral blood mononuclear cells (hPBMCs), approval was obtained from Institution Ethics Committee (IEC) of CCMB, Hyderabad, India (permit number IEC 35/2015). All reagents were purchased from Invitrogen unless otherwise specified. Cell lines including Chinese hamster ovary cells (CHO-K1ATCC, CCL-61), mouse embryonic stem (mES) cells-R1 (ATCC, SCRC-1011), and human embryonic kidney cells (HEK-293T/17ATCC, CRL-11268) were purchased from American Type Culture Collection (ATCC). The immortalized C18-4 mouse type-A spermatogonia cell line [33] was a gift from Dr. Marie-Claude Hofmann (The University of Texas MD Anderson Cancer Center, Houston, TX, USA). Human PBMCs were isolated as previously described [27] from the blood collected from healthy volunteers of either sex after obtaining written consent (n = 9). Both the MEF cells [34] and mBMCs [35] were derived from C57BL/6 mice (n = 6) as previously described. All adherent cells were cultured in Dulbecco's modified Eagle's medium (DMEM) with high glucose and suspension cells in Roswell Park Memorial Institute (RPMI) 1640 medium. Both media were supplemented with10% heat-inactivated FBS, 1 × non-essential amino acid solution, and 1 × antibiotic–antimycotic solution, and cells were cultured in a 5% CO_2_ environment at 37°C. Fresh confluent adherent cells after 24 h culture and fresh suspension cells after 72 h culture were considered as the control group.

### Cell cryopreservation

The uncontrolled slow freezing protocol was used for cell cryopreservation. Cryomedia for freezing consisted of DMEM/F12-HEPES that contained 10% DMSO (Sigma) alone (D10) and with 20% FBS (v/v) (D10S20) or 20% BuOF (v/v) (D10O20). In each 2-ml cryovial, cell suspension (1×10^7^cells) was added to 1ml of cryomedium and equilibrated for 30 min on ice. The cryovials were placed in an isopropyl alcohol container (Mr. Frosty Freezing Container; Thermo Scientific) and kept in a -80°C freezer (Thermo Scientific). Cells were cooled at an uncontrolled rate of approximately 1°C/min following the manufacturer’s protocols. After 24 h, cryovials were transferred to liquid nitrogen for storage.

### Cell thawing

After 1month, the cells were thawed by swirling the cryovials in a 37°C water bath until the contents were completely melted. The thawed content was transferred to a15-ml tube that contained 10-ml DMEM/F12-HEPES supplemented with 10% FBS. The content in the tube was gently mixed, centrifuged at 200 × *g* for 5 min, and the cell pellet was either suspended in DMEM/high glucose or RPMI 1640 medium with 10% FBS depending on cell type. Cell viability was determined before seeding the cells for culture.

### Cell viability and cell recovery assay

Cell viability was assessed immediately after thawing and after 24 h of culture (adherent cells) and 72 h culture (suspension cells). Cell viability was determined by trypan blue dye (Sigma) exclusion analysis. Microscopic evaluation was carried out no later than 10 min after the end of incubation. Approximately 500 cells/group were counted for cell viability analysis.

For cell recovery assay, the cells were seeded according to the post-thaw viability in each group. Adherent cells were cultured in 100-mm culture dishes and suspension cells in 75-mm^2^ culture flasks (both from TPP) at a density of 2 × 10^5^ live cells/cm^2^ using the above-mentioned medium and culture conditions. After 24 h, the adhered cells were washed twice with PBS, and the cells were harvested by trypsin (0.25%) digestion. The suspension cells were collected after 72 h of culture by pelleting down at 200 × *g* for 5 min. The recovered cells were assessed for viability and counted to estimate cell recovery [27] in each cryopreservation group.

### Western blot analysis

Frozen–thawed adherent cells cultured for 24 h and suspension cells cultured for 72 h were assessed for the expression of apoptosis and cell proliferation-specific proteins. Total proteins were extracted upon homogenization by sonication in a dissolving buffer (7M urea, 2M thiourea, 4% CHAPS [3-[(3-cholamidopropyl) dimethylammonio]-1-propanesulfonate], 18mM Tris-HCl, 14mM Tris-Base, 0.2% Triton-X, and 50mM dithiothreitol). Single strength ProteCEASE-50, which is an ethylenediaminetetraacetic acid (EDTA)-free protease inhibitor (G-Biosciences) was added to dissolving buffer before protein extraction. Lysed samples (30 μg) were subjected to 12% sodium dodecyl sulfate (SDS)-polyacrylamide gel electrophoresis (PAGE). The gels were transferred onto a polyvinylidenedifluoride (PVDF) membrane (Millipore). The membranes were blocked with Starting Block (TBS) Blocking Buffer (Life Technologies) for 1 h at room temperature. All primary and secondary antibodies were purchased from Thermo Scientificunless otherwise specified. The blocked membranes were incubated with the following primary antibodies to evaluate the expression of an early apoptotic protein, Annexin V, 1:1000 (Santa Cruz Biotechnology); a cell proliferation protein, proliferating cell nuclear antigen (PCNA), 1:1000; pro-apoptotic protein, BCL2-associated X protein (BAX), 1:200; and anti-apoptotic protein, B-cell lymphoma 2 (BCL2), 1:1000. To control protein loading on the gels, the membranes were probed with glyceraldehyde-3-phosphate dehydrogenase (GAPDH), 1:1000 antibody. The membranes were then washed with TBS-T and incubated in goat anti-mouse or goat anti-rabbit HRP-conjugated secondary antibody (1:10000) in TBS for 1 h at room temperature. After washing with TBS-T, immune reactivity was revealed by chemiluminescence using a C-DiGit Blot Scanner (Licor) against Super Signal West Femto Chemiluminescent Substrate (Thermo Scientific), and the generated signal was analyzed using a densitometer. Signal from each antibody was normalized to that of GAPDH for each cryopreservation group.

### Proteomic analysis

Total protein from BuOF and FBS samples was extracted using dissolving buffer (7M urea, 2M thiourea, 4% CHAPS, 18mM Trizma base, 2 tablets of EDTA protease inhibitor, 0.2% Triton X, and 50mM DTT). The extracted proteins were then quantified using the amido black assay [36]. One hundred micrograms of each sample in 2 × lamelli buffer (20% glycerol, 4% SDS, 10% 2-mercaptoethanol, 0.004% bromophenol blue, and 0.125M Tris HCl) was subjected to 12% SDS-PAGE in duplicate. The gel was stained in CBB R-250 (Coomassie brilliant blue R-250; Bio Rad) overnight and then de-stained and documented. Each of the electrophoresed sample lanes were divided into five fractions, and each fraction was further processed into finer, 1.5-mm pieces. Each of the fractions was washed in 40mM ammonium bicarbonate in 50% acetonitrile (ACN) for 1 hand twice with water for 1 h, followed by dehydration with 100% ACN. Each fraction was then digested for 18 h with 40μl of sequencing-grade trypsin (10 ng/μl; Promega). The digested peptides (100 μl) were extracted using 0.1% trifluoroacetic acid (TFA) in 50% ACN. The respective samples for each fraction were pooled, desalted, and then vacuum dried. The peptides were then reconstituted in 20 μl of 5% ACN and 0.1% formic acid. The trypsin-digested peptides were subjected to tandem mass spectrometry (MS/MS) using an Orbitrap Nano analyzer (Thermo Scientific). The samples were run in duplicate in collision-induced dissociation mode for 1 h each. The proteins were identified from the obtained MS/MS peak list searched against the *Bos taurus* database.

### Statistical analyses

Data were pooled from each trial for analysis. The results are presented as mean ± SEMof four trials for a cell type in each group. Statistical analysis was conducted using an analysis of variance (ANOVA). Significant differences between the means were determined by analyzing the data with the Fisher’s Least Significance Difference (LSD) test. The level of significance was set at P < 0.05.

## Results

### Biochemical compositions of BuOF and FBS

The biochemical compositions of BuOF and FBS are shown in Table 1. Most of the components were several-fold higher in FBS except for low-density lipoprotein (LDL) cholesterol and ascorbic acid, which were higher in BuOF.

### Post-thaw cell viability, cell recovery, and cell viability after culture

**Fig 1:**
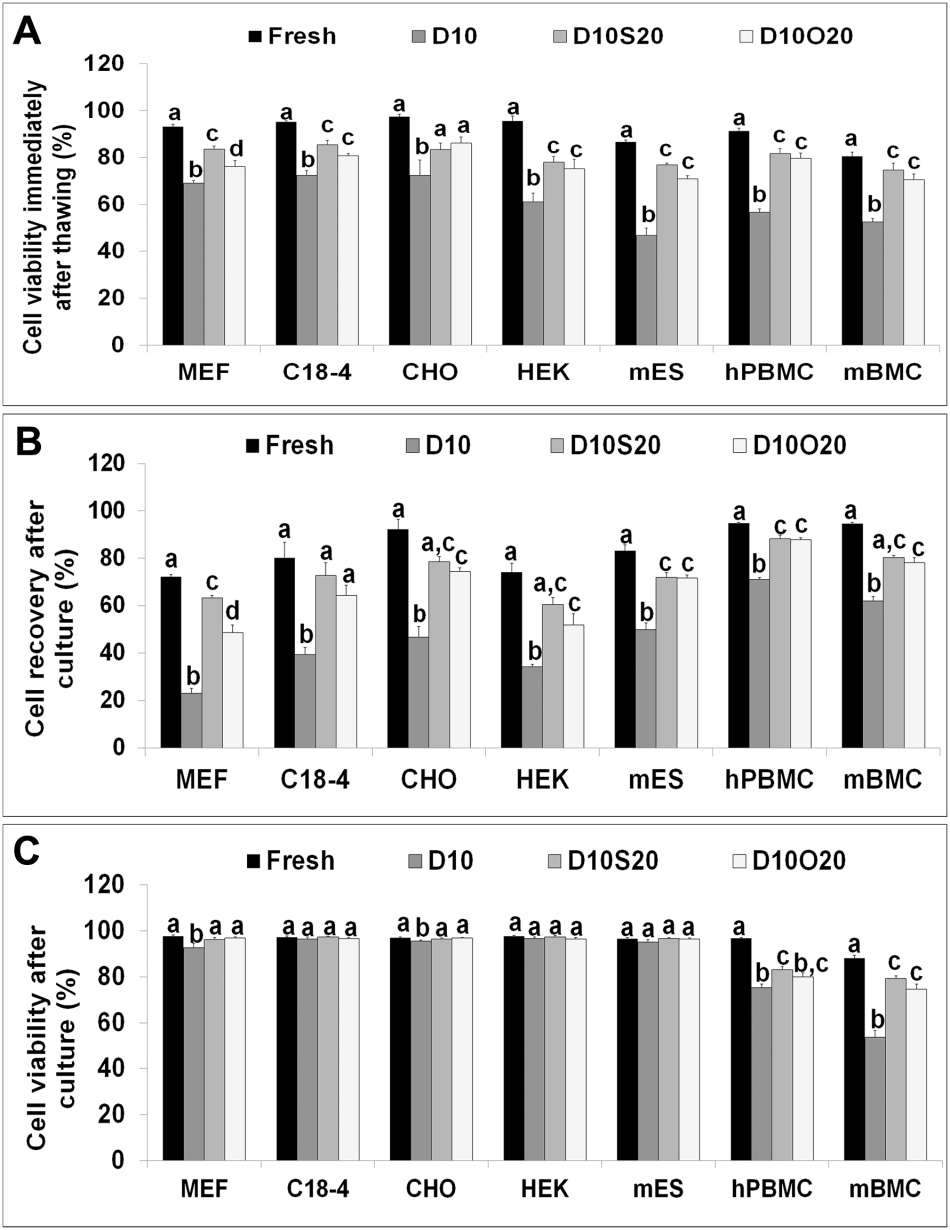
Cell viability and cell recovery in fresh and frozen–thawed cells. (A) Cell viability of fresh and frozen-thawed cells as determined by trypan blue dye exclusion immediately after thawing. (B) Percentage of cell recovery of frozen–thawed cells after 24 h (MEF, C18-4, CHO, HEK, and mES cells) and 72 h (hPBMCs and mBMCs) of culture. (C) Cell viability as determined by trypan blue dye exclusion after 24 h (MEF, C18-4, CHO, HEK, and mES cells) and 72h (hPBMCs and mBMCs) of culture. Data represent mean ± SEM off our trials for each cell type in each group. Bars with different letters are significantly different at P < 0.05.

Viability of cells was determined by trypan blue dye exclusion immediately after thawing (Fig 1A). The post-thaw viability of cells in all three adherent cell lines, mES cells, hPBMCs, mBMCs, and MEF cells frozen in D10 was significantly lower than that of fresh cells and cells frozen in D10S20 and D10O20. The viability of C18-4 cells, HEK cells, mES cells, hPBMCs, and mBMCs frozen in D10O20 was significantly lower than that of fresh cells but similar to that of cells frozen in D10S20. However, viability of CHO cells frozen in D10O20 was similar to that of both fresh cells and cells frozen in D10S20. Viability of MEF cells frozen in D10O20 was significantly lower than that of fresh cells and cells frozen in D10S20.

Frozen–thawed adherent cells 24 h and suspension cells 72 h after culture were evaluated to determine cell recovery (Fig 1B). There was a significant decline in cell recovery from D10, regardless of cell type; however, the cell recovery was similar in both D10S20 and D10O20 in all three adherent cell lines, mES cells, hPBMCs, and mBMCs. The recovery of C18-4 cells frozen in D10O20 was also similar to that of fresh cells. Fewer MEF cells were recovered in both D10S20 and D10O20 compared with fresh cells, and fewer were recovered in D10O20 compared with D10S20.

Cell viability of all cell types was also determined after culture (Fig 1C). Viability of C18-4, HEK, and mES cells was similar to that of fresh cells in all cryopreserved groups including D10. The viability of MEF cells, CHO cells, hPBMCs, and mBMCs frozen in D10 was significantly lower than that of fresh cells and cells frozen in D10S20 and D10O20. The viability of hPBMCs and mBMCs frozen in D10O20 was significantly lower than that of fresh cells but similar to that of cells frozen in D10S20.

### Western blot analysis of frozen–thawed-cultured cells

**Fig 2:**
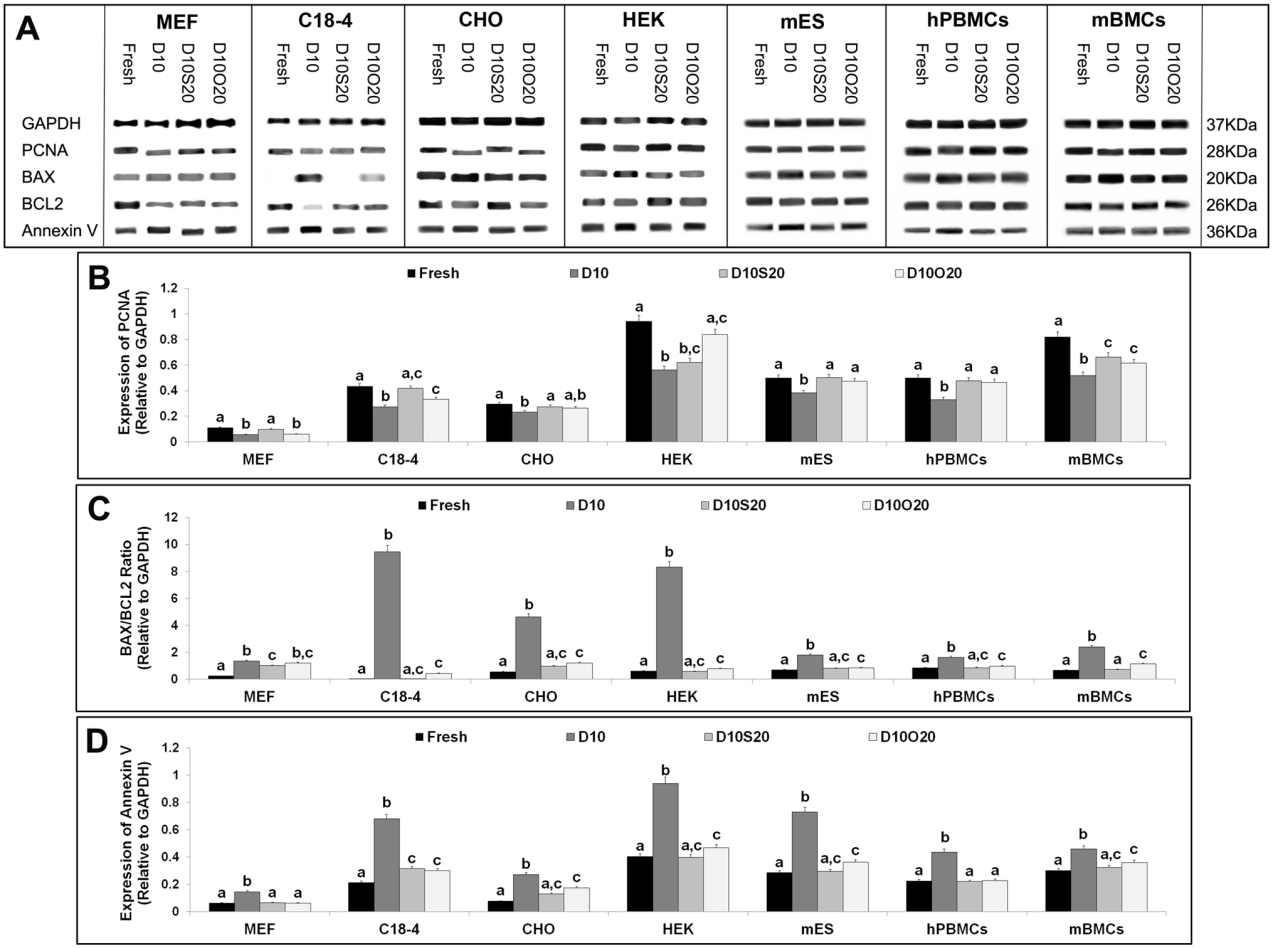
Expression of proliferation-, pro-apoptosis-, anti-apoptosis-, and early apoptosis-specific proteins in frozen-thawed cells. Expression of cell PCNA, BAX, BCL2, and Annexin V respectively in fresh and frozen–thawed cells after 24 h (MEF, C18-4, CHO, HEK, and mES cells) and 72 h (hPBMCs and mBMCs) of culture. Thirty-microgram aliquots of each cell extract were subjected to SDS-PAGE and western blot analysis. (A) Representative western blotting and densitometry analysis of (B) BAX/BCL2 ratio, (C) PCNA, and (D) Annexin V expression relative to GAPDH. Data represent mean ± SEM off our trials for a cell type in each group. Bars with different letters are significantly different at P < 0.05.

Expression levels of cell proliferation-, early apoptosis-, pro-apoptosis-, and anti-apoptosis-specific proteins (PCNA, Annexin V, BAX, and BCL2, respectively) were analyzed in frozen–thawed cells after culture (Fig 2A). PCNA expression in all cell types cryopreserved in D10 was lower than that in fresh cells and cells cryopreserved in D10S20 and D10O20 (Fig 2B). PCNA expression in CHO cells, hPBMCs, and mES cells frozen in D10O20 was similar to that of fresh cells and cells frozen in D10S20. PCNA expression in hPBMCs and C18-4 cells frozen in D10O20 was similar to those cells frozen in D10S20 but lower than that in fresh cells. In MEF cells, PCNA expression was lower in cells frozen in D10O20 than in both fresh and cells cryopreserved in D10S20. Interestingly, PCNA expression in HEK cells frozen in D10O20 was similar to that of fresh cells but higher than that of cells frozen in D10S20.

The ratio of BAX/BCL2 proteins was significantly higher in all cell types frozen in D10 than in fresh cells and cells cryopreserved in D10S20 and D10O20 (Fig 2C). Although the BAX/BCL2 ratio was higher in D10O20 compared with fresh cells, it was similar to that of cells frozen in D10S20 in all cell types except mBMCs. mBMCs frozen in D10O20 had higher BAX/BCL2 ratios compared with those frozen in D10S20. Annexin V protein expression in all seven cell types cryopreserved in D10 was higher than in fresh cells and cells cryopreserved in D10S20 and D10O20 (Fig 2D). Annexin V expression in MEF cells, and hPBMCs frozen in D10O20 was similar to that of fresh cells and cells frozen in D10S20. Annexin V expression in C18-4 cells, HEK cells, CHO cells, mES cells, and mBMCs frozen in D10O20 was similar to that of cells frozen in D10S20 but lower than that of fresh cells.

### Proteomic analysis of BuOF and FBS

Proteomic analysis of BuOF and FBS was carried out by depleting the samples of abundant proteins and subjecting the samples to MS/MS analysis. Based on MS/MS peak search in the *Bos taurus* database, a total of 41 proteins in BuOF and 207 proteins in FBS were identified, out of which 16 were common. A list of proteins identified in BuOF and their comparison with FBS is shown in Table 2. Proteins identified in FBS are listed in S1 Table. A protein database search and literature search revealed that BuOF contains 26% glycoproteins, 9% globular proteins, 7% plasma proteins, 9% lipoproteins, and 7% cytoskeletal proteins. Out of the 16 common proteins, albumin was the major protein identified in FBS and BuOF.

**Table 2:** Proteomic analysis of BuOF and comparison with FBS.

	Name of the protein	Molecular weight (kDa)	Type of protein/role	Whether present or absent in FBS
1	Apolipoprotein A-I precursor	30.3	High density lipoprotein/promotes proliferation and inhibits apoptosis of cells [77]	Present
2	Fibronectin variable region	20.4	Glycoprotein/induces cell proliferation, inhibits apoptosis [83], and helps improve fertility rate of cryopreserved sperm [84]	Present
3	Transthyretin precursor	15.7	Thyroxine binding prealbumin	Present
4	Serum albumin	53.9	Globular protein/replaces serum in efficient cryopreservation of PBMCcells [27]	Present
5	Alpha-1-B glycoprotein precursor	53.5	Glycoprotein	Present
6	Gelsolin	80.7	Lysophosphatidic acid transport protein/anti-oxidant properties [73] andinvolved in both the control and execution of apoptosis [72]	Present
7	Complement component 3	187.1	Glycoprotein	Present
8	Keratin 5, type II	62.6	Cytoskeletal protein	Present
9	Inter-alpha-trypsin inhibitor heavy chain H2 precursor	106.1	Carrier protein	Present
10	Albumin	69.2	Globular protein/replace sserum in efficient cryopreservation of PBMC cells [27]	Present
11	Complement factor B precursor	85.3	Circulatory protein	Present
12	Keratin 7, type II	50	Cytoskeletal protein	Present
13	Transferrin isoform X1	77.6	Glycoprotein/major anti-oxidant protein andprotects spermatozoa against oxidative damage during freeze–thawstress [69]	Present
14	Keratin 42, type 1 like isoform X3	50.3	Cytoskeletal protein	Present
15	Serpin peptidase inhibitor, clade A (alpha-1 antiproteinase, antitrypsin), member 1 isoform X1	46.1	Enzyme/inhibit sapoptosis by inhibitingcaspase-3 [75]	Present
16	Crystal structure of BSA chain b	66.4	Globular protein/replaces serum in efficient cryopreservation of PBMCcells [27]	Present
17	Alpha-2-HS-glycoprotein precursor	38.4	Glycoprotein	Absent
18	Serpin peptidase inhibitor, clade A (alpha-1 antiproteinase, antitrypsin), member 3	22.7	Globulin glycoprotein/inhibits apoptosis by inhibiting caspase-3 [75]	Absent
19	Regucalcin	33.3	Calcium binding protein/maintains cell homeostasis and playa role as a suppressor protein in cell signaling systems in many cell types [81]	Absent
20	Crystal structure of bovine factor Vai, chain A	34.7	Inactivated form of factor Va	Absent
21	Endopin 2C	46.7	Type of serpin/inhibits cysteine proteases and elastase-like serine proteases therefore eventually inhibit apoptosis [85]	Absent
22	Fetuin B precursor	42.6	Glycoprotein/anti-oxidant and helps maintain sperm morphology by increasing ROS scavenging enzymes [82]	Absent
23	Fibrinogen alpha chain	18.1	Glycoprotein	Absent
24	Apolipoprotein E	27.1	Lipoprotein	Absent
25	C-type lectin domain family 3, member B precursor	22.1	Plasminogen binding protein	Absent
26	Adiponectin	26.1	Hormone(cytokine)	Absent
27	Transferrin precursor	77.7	Glycoprotein/major anti-oxidant protein andprotects spermatozoa against oxidative damage during freeze–thaw stress [69]	Absent
28	Inter-alpha-trypsin inhibitor heavy chain 1 precursor	101.2	Serine protease inhibitor	Absent
29	keratin 25, type I like	49.3	Intermediate filament	Absent
30	Bovine hemoglobin chain C	15	Metalloprotein	Absent
31	Hemoglobin, beta	16	Globin protein	Absent
32	Apolipoprotein N	28.5	Lipoprotein	Absent
34	Collagen, type III, alpha 1	137.1	Fibrous scleroprotein	Absent
35	Immunoglobulin light chain	10.4	Polypeptide	Absent
36	Alpha-2-macroglobulin variant 1	115.1	Plasma protein/proteinase inhibitor and anti-apoptotic protein [86]	Absent
37	Alpha-2-macroglobulin variant 4	42.3	Plasma protein/proteinase inhibitor and anti-apoptotic protein [86]	Absent
38	Kininogen isoform X2	44.4	Polypeptide	Absent
39	Protein dimmed	39.4	Helix-loop-helix protein	Absent
40	Primary amine oxidase	81.7	Copper containing enzymes/biological regulator of cell growth and differentiationand is also involved in apoptosis regulation by altering membranes [78]	Absent
41	Actin, alpha 2, smooth muscle, aorta	39.3	Globular protein	Absent
42	Methylenetetrahydrofolate dehydrogenase (NADP+ dependent) 1-like	81.6	Ligase	Absent

## Discussion

Because FBS is enriched with several growth factors and proteins, it is routinely used as a media supplement for cell and tissue cultures. FBS is also used as a non-penetrating cryoprotectant to cryopreserve several cell types. High cost, ethical concerns, and the possibility of transmission of blood-borne diseases has led to a need to find a substitute for cryopreservation [22]. To date, many serum replacements have been introduced for cell cryopreservation [25–27]; however, their use is limited, primarily because of their high cost and difficulty in procurement. In the present study, we investigated if BuOF can replace FBS for cryopreservation of several different cell types.

The biochemical composition of BuOF was found to be different from that of FBS. All constituents except for LDL cholesterol and ascorbic acid, were found to be present in a higher concentrations in FBS (approximately 1.8- and 6.4-fold higher in BuOF, respectively; Table 1). LDL cholesterol contains 85–90% lipids as well as 10–15% proteins [37] and is responsible for the gelation process in freeze–thawing [37–40]. Presence of higher LDL cholesterol content in BuOF could be beneficial for cell cryopreservation by providing fluid stability. LDL cholesterol protected sperm cells against cold shock by preventing the efflux of phospholipids and cholesterol from the sperm cell membrane [41]. Egg yolk, which is rich in LDL cholesterol, has been proven to be an effective cryopreservative for spermatozoa of bulls [42, 43], stallions [44], rams [45], and dogs [46].

Ascorbic acid is a micronutrient that prevents both membrane depolarization and cytochrome C release events that occur during apoptosis [47]. Lane *et al*. observed a significant decrease in the levels of lactate dehydrogenase in mouse embryos cryopreserved with ascorbic acid [48]. Presence of a high concentration of ascorbic acid in BuOF could have a role in preventing damage by free radicals, which is one of the reasons implicated in loss of viability during or immediately after cell freezing [49]. Thus, viability of different cell lines was maintained in this study. Furthermore, a high concentration of ascorbic acid in BuOF could be responsible for the similarity in Annexin V expression and BAX/BCL2 ratio in cell lines and hPBMCs cryopreserved in BuOF and FBS. However, it remains unclear if the effect was exclusively because of ascorbic acid or some other components in BuOF.

Annexin V expression has been used to evaluate early apoptotic cells [50], whereas the ratio of BAX (pro-apoptotic) to BCL2 (anti-apoptotic) gene expression indicates susceptibility of cryopreserved cells to apoptosis [51]. Previous study revealed that Annexin V assay can be used to detect membrane integrity of frozen–thawed human spermatozoa [52]. Moreover, the BAX/BCL2 ratio is used to determine the quality of embryos and oocytes in different stages of *in vitro* culture [53]. The relative Annexin V expression and BAX/BCL2 ratio were similar in all cell types cryopreserved in either D10S20 or D10O20except mBMCs and in cells frozen in D10. These findings indicate that both FBS and BuOF have similar ability to inhibit apoptosis in most cell types cryopreserved in this study. Although in mouse primary suspension cells such as mBMCs frozen in D10O20, the BAX/BCL2 ratio was similar to that of cells frozen in D10. Nevertheless, Annexin V expression in mBMCs cryopreserved in either group was not different. These findings indicate that unlike BuOF, FBS was beneficial for preventing late apoptosis in mBMCs. Interestingly, Annexin V expression and BAX/BCL2 ratio in human primary cells (hPBMCs) was similar in both groups. These results suggest that mBMCs are rather susceptible to apoptosis during cryopreservation, unlike hPBMCs. The components of the p53 pathway that control late cellular apoptosis in response to DNA damage are reported to be more active in mouse cells than in human cells [54, 55]. This could potentially explain the elevated BAX/BCL2 ratio in mouse primary cells.

Recovery of viable cells from cryogenic storage is a major concern [56]. Initially, viability of all cell types was estimated immediately after thawing to compare BuOF and FBS cryopreservation abilities, as reported in earlier studies [26, 27, 57]. Although viability of MEF cells frozen in D10O20 immediately after thawing was low, expression of Annexin V and BAX/BCL2 ratio in cultured cells were not different from cells frozen in D10S20. These findings suggest that though BuOF could not preserve the initial viability nevertheless, it could prevent both early and late apoptosis of frozen-thawed MEF cells. On the contrary, all cells types frozen in D10 not only had lower cell viability immediately after thawing but also had elevated Annexin V and BAX/BCL2 ratio in cultured cells. These findings indicate that presence of FBS or BuOF in cryomedia significantly enhances cell survival and prevented apoptosis. Interestingly, the cell viability of few cultured adherent cells such as C18-4, HEK and mES cells that were frozen in D10 did not differ from cells frozen in other groups, but the Annexin V and BAX/BCL2 ratio in these cells were significantly higher. These results indicate that mere recovery of cell viability of frozen-thawed cells cannot be a reliable indicator for the cryoprotection provided by a cryopreservation media [58]. Evaluation of apoptosis-specific proteins expression in frozen-thawed cells is also essential [59]. Because different cells grow at different rates, cell recovery post-culture is an ideal method to assess their growth rate after cryopreservation. Recovery of cells can be assessed by percentage of cell recovery [60] and expression of proliferation cell-specific proteins, such as PCNA [61]. Frozen–thawed murine neural precursor cells retained their proliferative capacity, as shown by the PCNA expression [62]. Percentage of cells recovered and PCNA expression in cells frozen in DMSO alone (D10) was significantly lower than that of cells frozen with DMSO with FBS or BuOF in all cell types used in this study. These findings further support the use of FBS or BuOF in cryomedia for cell cryopreservation.

However, for the MEF cells cryopreserved in D10O20, cell recovery and PCNA expression were significantly lower than that in the cells cryopreserved in D10S20, and the viability of cultured cells recovered after 24h was not different from fresh cells and cells frozen in D10S20. This difference could be because MEF cells are primary cells and require more than 24 h for restoration of cell growth and proliferation after cryopreservation. Another plausible explanation could be presence of higher protein, lipoprotein, and triglyceride contents in FBS led to better cryopreservation of MEF cells. Bradykinin [63], tubulin [64], heat shock protein 90 [65], and superoxide dismutase [66] are reported to play roles in bull sperm cryopreservation. Enolase, a metalloenzyme, could replace DMSO in cryopreservation of rat hepatocytes by reducing oxidative stress and cellular toxicity [67]. Presence of these proteins exclusively in FBS could potentially provide superior cryoprotection.

Although the protein content of BuOF was much lower than that of FBS, a comparative proteomic analysis of BuOF and FBS was carried out to identify proteins/peptides that may be critical for cryopreservation. A total of 41 proteins were identified in BuOF through MS/MS analysis. The majority of proteins were glycoproteins, globular proteins, and lipoproteins. Out of the 41 identified proteins, 16 were also present in FBS (Table 2). Transport proteins such as albumin, serotransferrin, transthyretin, and apolipoprotein AI that were present in both FBS and BuOF were previously reported in human vitreous humor [68], which validates the proteomic approach that was followed in this study.

These proteins are biologically essential, have important roles in cell homeostasis, and act as hormone carriers. Transferrin protects spermatozoa against oxidative damage during freeze–thaw stress [69]. Fibronectin, which is another important glycoprotein that was identified in BuOF, plays an important role in cell–cell aggregation, cell–substratum adhesion, attachment of cells to extracellular matrix components, and cellular motility [70]. Gelsolin, which is an actin filament that caps and severs proteins that enhance the rate of cell migration [71], is involved in both the control and execution of apoptosis [72]. This lysophosphatidic acid transport protein is thought to have anti-oxidant properties [73]. The presence of these proteins in BuOF could be a reason for lower expression of pro-apoptotic proteins in cryopreserved cell lines.

Several proteolytic enzymes have been implicated in apoptosis and associated processes. α-1 Antiproteinase (A1P1) in BuOF is a serine protease inhibitor (serpin) [74] that regulates apoptosis by inhibiting caspase-3 activity [75]. Many proteins identified in BuOF and FBS were found to be involved in lipid transport and binding as well as cell migration. Transthyretin, which is a transport protein in BuOF, is involved in lipid metabolism [76]. Apolipoprotein A-I, which is a major component of high-density lipoprotein (HDL), has been reported to promote proliferation and inhibit apoptosis of endothelial and vascular smooth muscle cells [77]. Presence of these proteins in BuOF may be critical for preventing cell apoptosis during freezing and thawing.

A few proteins were exclusively identified in BuOF. Amine oxidase and kininogen are reported to play crucial role in prevention of cellular apoptosis [78, 79]. Actin plays a crucial role in cell structure, motility, and cell division [77]. Damage to actin modifies microtubule organization in the presence of cryoprotectants such as propanediol and DMSO [80]. The globular and glycoproteins present in BuOF contribute to important cellular functions, such as mobility and contraction of cells during cell division. Regucalcin has a role as a suppressor protein in cell signaling systems of many cell types [81]. Fetuin, a glycoprotein and protease inhibitor, increases superoxide dismutase and glutathione peroxidase enzymatic activities to minimize membrane and DNA damage in cryopreserved semen [82]. Whether these proteins have a critical role in cell cryopreservation needs to be investigated further.

This is a preliminary study that identified BuOF as a replacement for FBS in cryopreservation media. The composition of ocular fluid may vary with age, sex, breed, origin, and physiological and health status of the animal. Variation in the composition of ocular fluid may affect cell survival and gene expression of cryopreserved cells. Therefore, further investigations and standardizations are needed before BuOF can find its use for commercial applications.

In conclusion, this study demonstrates efficient cryopreservation of adherent cell lines, such as CHO, HEK, C18-4, and mES cells and primary suspension cells such as hPBMC and mBMC in 20% BuOF, and its potential to replace FBS in cryomedia. Because mouse primary cells (mBMCs and MEF cells) are susceptible to cryodamage, cryopreservation using BuOF needs further investigation. Proteomic and biochemical analyses of BuOF identified several components that may have crucial roles in cell cryopreservation. Further studies that elucidated the effect of BuOF in several different cell types could help establish BuOF as a vital component for serum-free cryopreservation protocols.

## Supporting Information

S1 Table Proteomic analysis of fetal bovine serum (FBS). (DOCX)
