# Supplementary material for: Ocular Fluid As a Replacement for Serum in Cell Cryopreservation Media
Source: PLoS One. 2015 Jul 2;10(7):e0131291. doi: 10.1371/journal.pone.0131291 (PMC4489643; doi:10.1371/journal.pone.0131291)
Supplement: S1 Table — (DOCX) [file pone.0131291.s001.docx]

**S1. Supplementary tables 1: Proteomic analysis of fetal bovine serum (FBS)**

|  | **Name of the protein** | **Molecular weight (KDa)** | **Type of protein** |
| --- | --- | --- | --- |
|  | Crystallin, beta A1 | 25.1 | Structural protein |
|  | Crystallin, beta A1 subunit | 13.1 | Structural protein |
|  | Retinol binding protein, [interstitial](http://www.genenames.org/cgi-bin/gene_symbol_report?hgnc_id=HGNC:9921) | 39.6 | Glycoprotein |
|  | Bradykinin | 1.1 | Nonapeptide/ improves the quality of cryopreserved semen [[1](#_ENREF_1)] |
|  | Beaded filament structural protein 2, phakinin (bovine) | 45.5 | Intermediate filament |
|  | Crystallin, gamma B | 10.3 | Structural protein |
|  | Aldehyde dehydrogenase 3 family, member A1 | 26.7 | Enzyme |
|  | Tyrosine 3-monooxygenase/tryptophan 5-monooxygenase activation protein, beta | 27.8 | Enzyme/suppresses several apoptotic pathways [[2](#_ENREF_2),[3](#_ENREF_3)] |
|  | Crystallin, gamma S c-terminal domain | 10.3 | Structural protein |
|  | Versican V3 splice-variant precursor | 74.7 | Proteoglycan |
|  | Crystallin, zeta | 35.4 | Structural protein |
|  | AE binding protein 1 | 82.3 | DNA binding protein |
|  | Chaperonin containing TCP1, subunit 8 (theta), partial | 19.8 | Protein chaperone |
|  | Heat shock 70KDa, chain A | 41.2 | Chaperone protein cytoprotective properties explained by its anti-apoptotic function [[4](#_ENREF_4)] |
|  | Fibronectin variable region | 20.4 | Glycoprotein |
|  | Creatine kinase, bovine retinal chain a | 42.6 | Enzyme |
|  | Crystallin, gamma A (c18s mutant of bovine) | 20.9 | Structural protein |
|  | Filamin A interacting protein 1, partial | 53.1 | Globular protein/inhibits apoptosis induced by the mechanical forces [[5](#_ENREF_5)] |
|  | Crystallin, alpha B | 20.0 | Structural protein |
|  | Crystallin, alpha A (Bos taurus) | 19.8 | Structural protein |
|  | Contactin 1, precursor | 113.3 | Membrane protein |
|  | Beaded filament structural protein 2, phakinin (Bos taurus) | 45.9 | Lens structural protein |
|  | Retinal dehydrogenase 1 | 54.8 | Enzyme |
|  | Ezrin | 68.7 | Membrane protein |
|  | Retinol-binding protein 3 (precursor) | 139.6 | Carrier protein |
|  | Serpin peptidase inhibitor, clade F (alpha-2 antiplasmin, pigment epithelium derived factor), member 1 (precursor) | 46.2 | Secreted protein/plays anti-apoptotic role by inhibiting lysosomal degradation of Bcl-xL [[6](#_ENREF_6)] |
|  | Protein disulfide isomerase family A, member 6 (precursor) | 57.2 | Enzyme |
|  | Transthyretin (precursor) | 15.7 | Thyroxine binding pre-albumin |
|  | Clusterin preproprotein | 51.1 | Glycoprotein/inhibits apoptosis by interacting with activated Bax [[7](#_ENREF_7)] |
|  | Crystallin, beta A2 | 22.2 | Structural protein |
|  | Crystallin, beta B1 | 28.1 | Structural protein |
|  | Phosphatidylinositol-specific phospholipase D (precursor) | 92.5 | Enzyme |
|  | Spondin 1, extracellular matrix protein (precursor) | 90.9 | Secreted protein |
|  | Elongation factor 1 alpha | 13.0 | Enzymatic delivery of aminoacyl tRNAs to the ribosome and nuclear export of proteins |
|  | Tubulin, alpha 1 | 10.4 | Globular protein/binds to dynein in sperm and forms cold resistant microtubules [[8](#_ENREF_8)] |
|  | Elongation factor 1 gamma | 17.2 | Enzymatic delivery of aminoacyl tRNAs to the ribosome |
|  | Heat shock protein 90KDa beta, partial | 27.8 | Chaperone protein |
|  | Glutamic-oxaloacetic transaminase 2, cytoplasmic | 46.4 | Enzyme |
|  | Transketolase | 9.6 | Enzyme |
|  | Crystallin, beta A4 | 22.5 | Structural protein |
|  | Decorin, 2b | 36.3 | Proteoglycan |
|  | Creatine kinase, muscle | 43.0 | Enzyme |
|  | Heat shock protein 90KDa alpha | 84.7 | Chaperone protein protect cells from ROS [[9](#_ENREF_9)], involved in freezing resistance of bull sperm [[10](#_ENREF_10)] |
|  | UDP-GlcNAc:betaGal beta-1,3-N-acetylglucosaminyltransferase 6 | 43.8 | Enzyme |
|  | Rab geranylgeranyltransferase, alpha subunit | 38.1 | Enzyme |
|  | Crystallin, gamma C | 20.9 | Structural protein |
|  | Triosephosphate isomerase | 26.7 | Enzyme |
|  | Complement factor B, partial | 9.1 | Glycoprotein |
|  | Albumin, partial [Bos indicus] | 53.9 | Globular protein/replaces FBS for cryopreservation of cells [[11](#_ENREF_11)] |
|  | Phosphoglycerate kinase 1 | 44.5 | Enzyme |
|  | Secreted frizzled-related protein 2 precursor | 33.3 | Glycoprotein |
|  | Carbonic anhydrase III | 29.4 | Enzyme |
|  | NSFL1 (p97) cofactor (p47) | 40.6 | ATPase |
|  | Tubulin, alpha 4a | 49.6 | Globular protein |
|  | Enolase 3 (beta, muscle) | 47.1 | Enzyme/efficiently replaces DMSO for rat hepatocytes cryopreservation without cellular toxicity [[12](#_ENREF_12)] |
|  | Phosphorylase, glycogen; brain | 96.3 | Enzyme |
|  | GPD1, partial | 37.0 | Enzyme |
|  | Glyceraldehyde-3-phosphate dehydrogenase | 24.2 | Enzyme |
|  | Vimentin | 53.7 | Intermediate filament protein |
|  | Selenium binding protein 1 | 52.5 | Enzyme |
|  | Leucine-rich alpha-2-glycoprotein 1 precursor | 38.3 | Glycoprotein |
|  | Keratin 7, type II | 51.5 | Cytoskeletal protein |
|  | Protein HP-25 homolog 2 precursor | 22.9 | Hibernation specific plasma protein |
|  | Alpha-1-B glycoprotein precursor | 53.5 | Glycoprotein |
|  | Glyceraldehyde-3-phosphate dehydrogenase pseudogene 2 | 35.8 | Enzyme |
|  | Phosphoglucomutase 5 partial | 45.1 | Enzyme |
|  | Gelsolin | 80.7 | Lysophosphatidic acid transport protein/control and execution of apoptosis [[13](#_ENREF_13)] with anti-oxidant properties [[14](#_ENREF_14)] |
|  | Phosphofructokinase, liver | 85.2 | Enzyme |
|  | Complement component 3 | 187.1 | Glycoprotein |
|  | Keratin 5, type II | 62.6 | Cytoskeletal protein |
|  | Fibulin-1 precursor | 77.5 | Calcium binding glycol protein |
|  | Ribonuclease L (2',5'-oligoisoadenylate synthetase-dependent) | 81.7 | Enzyme |
|  | Inter-alpha-trypsin inhibitor heavy chain 2 precursor | 106.1 | Carrier protein |
|  | CAP, adenylate cyclase-associated protein, 2 | 53.7 | Enzyme |
|  | Uridine phosphorylase 1 | 33.9 | Enzyme |
|  | Dickkopf WNT signaling pathway inhibitor 3precursor | 38.4 | Secreted protein |
|  | Albumin | 69.2 | Globular protein |
|  | Fumarylacetoacetate hydrolase (fumarylacetoacetase) | 46.1 | Enzyme |
|  | Quiescin Q6 sulfhydryl oxidase 1 | 62.9 | Enzyme |
|  | Biotinidase precursor | 58.3 | Enzyme |
|  | FK506 binding protein 1A, 12KDa | 11.8 | Enzyme |
|  | Leucine aminopeptidase 3 (unligated) | 52.6 | Enzyme |
|  | Crystallin, beta A1 | 20.7 | Structural protein |
|  | Alpha-2-macroglobulin precursor | 167.5 | Globular protein/proteinase inhibitor and anti-apoptotic protein [[15](#_ENREF_15)] |
|  | Secernin 1 | 46.1 | Exocytosis regulating protein |
|  | Heat shock 70KDa protein 4 | 94.4 | Chaperone protein |
|  | Tubulin-colchicine-phomopsin A, chain D | 49.9 | Globular protein |
|  | Phosphogluconate dehydrogenase | 53.0 | Enzyme |
|  | Keratin 14, type I | 51.8 | Cytoskeletal protein |
|  | Kinesin family member 5C | 109.1 | Motor protein |
|  | Crystal structure of human STAM1 VHS domain in complex with ubiquitin, chain g | 8.3 | Trafficking protein |
|  | Ca Model Of Bovine TricCCT DERIVED FROM A 4.0 ANGSTROM Cryo-Em Map, chain Q | 55.7 | Chaperone protein |
|  | Crystallin, alpha A (bovine) | 11.6 | Structural protein |
|  | Transcriptional regulator ICP4 | 141.7 | Nuclear protein |
|  | Filamin A, alpha | 275.4 | Globular protein/protect the cells from force induced apoptosis [[16](#_ENREF_16)] |
|  | Nucleosome assembly protein 1-like 4 | 44.0 | Interact with both core and linker histones |
|  | Actinin, alpha 2, partial | 99.3 | Actin binding protein regulate cell survival by TNF-alpha-induced survival signalling [[17](#_ENREF_17)] |
|  | Sortilin-related VPS10 domain containing receptor 1 | 129.9 | Associates with serum lipoproteins |
|  | T-complex 1, zeta | 53.5 | Molecular chaperone |
|  | Complement factor B precursor | 85.3 | Glycoprotein |
|  | Heat shock protein 90KDa beta | 78.6 | Chaperone protein/protect cells from ROS [[9](#_ENREF_9)], involved in freezing resistance of bull sperm [[10](#_ENREF_10)] |
|  | Endopin 1b-like | 26.3 | Serpin protease inhibitors/inhibits cysteine proteases and elastase-like serine proteases therefore, eventually inhibiting apoptosis [[18](#_ENREF_18)] |
|  | Tight junction protein (ZO) 1 | 144.0 | Transmembrane protein |
|  | EGF-like, fibronectin type III and laminin G domains | 99.6 | Glycoprotein |
|  | Keratin 10, type I | 54.5 | Cytoskeletal protein |
|  | Oligodendrocyte myelin glycoprotein | 49.4 | Glycoprotein |
|  | Protein phosphatase 2, regulatory subunit A, alpha | 65.2 | Enzyme |
|  | Hemicentin 1 | 612.2 | Immunoglobulin superfamily |
|  | Tripeptidyl peptidase I precursor | 61.2 | Enzyme |
|  | Apolipoprotein A-I like | 23.6 | High density lipoprotein/promote proliferation and inhibits apoptosis of cells [[19](#_ENREF_19)] |
|  | Fatty acid binding protein 5, epidermal | 11.9 | Intracellular binding protein |
|  | N-myc downstream regulated 1 | 41.6 | Helix-loop-helix protein |
|  | RAN binding protein 6 | 47.3 | Nuclear transport receptor |
|  | CAP, adenylate cyclase-associated protein 1 like | 51.3 | Enzyme |
|  | BRCA1 cofactor | 87.6 | Induce large-scale chromatin reorganization |
|  | Actin, gamma 2, smooth muscle, enteric | 39.7 | Globular protein |
|  | Malic enzyme 1, NADP(+)-dependent, cytosolic | 58.9 | Enzyme |
|  | ATPase, class V (ER) | 89.2 | Enzyme |
|  | Talin 1 | 269.7 | Actin binding protein |
|  | Ankyrin 2, neuronal like | 433.4 | Integral membrane protein |
|  | ABI family, member 3 (NESH) binding protein | 39.3 | Enzyme |
|  | Keratin 1, type II | 63.1 | Cytoskeletal protein |
|  | Eukaryotic translation initiation factor 3, subunit F pseudogene 2 | 35.6 | Involved in initiation of translation |
|  | Unnamed protein product | 47.2 |  |
|  | Karyopherin (importin) beta 1 | 97.2 | Protein involved in transport of molecules between cytoplasm and nucleus |
|  | Clathrin, heavy chain | 186.8 | Involved in membrane traffic pathways |
|  | Dynein, cytoplasmic 1, heavy chain 1 | 531.6 | Motor protein |
|  | NAD(P)H dehydrogenase, quinone 2 | 55.5 | Enzyme |
|  | Keratin 1b, type II | 50.0 | Cytoskeletal protein |
|  | Protein tyrosine phosphatase, receptor-type, Z polypeptide 1 | 168.4 | Enzyme |
|  | RNA polymerase, sigma-24 subunit | 22.8 | Enzyme |
|  | Ceruloplasmin (ferroxidase) precursor | 120.7 | Enzyme |
|  | Model Refined Against Symmetry-free Cryo-em Map Of Tric-adp, Chain P | 55.0 | Chaperone protein |
|  | Glucose-6-phosphate isomerase | 62.8 | Enzyme |
|  | Profilin, chain A | 41.6 | Actin binding protein |
|  | Group-specific component (vitamin D binding protein) | 53.3 | Globular protein |
|  | Thioredoxin | 11.8 | Enzyme |
|  | Alpha-2-macroglobulin variant 22 | 77.3 | Globular protein/proteinase inhibitor and anti- apoptotic protein [[15](#_ENREF_15)] |
|  | Heat shock 70KDa protein, partial | 69.4 | Chaperone protein |
|  | Hypothetical protein A7H1H_1263 | 38.4 | Globular protein |
|  | tRNA methyltransferase 5 | 57.2 | Enzyme |
|  | Contactin 4 | 113.2 | Membrane protein |
|  | Fibulin 2 | 124.0 | Calcium binding glycol protein |
|  | DCC netrin 1 receptor | 141.1 | Transmembrane protein |
|  | Complement component 4A | 187.7 | Glycoprotein |
|  | Amyloid beta (A4) precursor protein-binding | 76.6 | Adaptor protein localized in the nucleus |
|  | Crystallin, beta S | 20.9 | Structural protein |
|  | Eukaryotic translation initiation factor 4A1 | 45.0 | Enzyme |
|  | Transferrin | 77.6 | Glycoprotein |
|  | Fibronectin | 239.3 | Glycoprotein/induce cell proliferation, inhibit apoptosis [[20](#_ENREF_20)], improves fertility rate of cryopreserved sperm [[21](#_ENREF_21)] |
|  | Glutathione S-transferase mu 1 | 21.1 | Enzyme |
|  | Neuronal cell adhesion molecule | 130.8 | Glycoprotein |
|  | Filamin C | 277.2 | Globular protein |
|  | Lumican | 38.7 | Proteoglycan/promote cell proliferation and regulates apoptosis [[22](#_ENREF_22)] |
|  | Keratin 2, type II | 64.1 | Cytoskeletal protein |
|  | Tubulin, alpha 1c | 46.0 | Globular protein |
|  | Cullin-associated and neddylation-dissociated 1 | 123.8 | Enzyme |
|  | Eukaryotic translation initiation factor 3, subunit L | 61.0 | Initiation of protein synthesis |
|  | Hsc70-interacting protein isoform X1 | 40.2 | Chaperone protein |
|  | Protein kinase C and casein kinase substrate in neurons 2 | 51.4 | Enzyme |
|  | Plexin B2 | 204.8 | Semaphorin membrane receptor protein |
|  | Ankyrin 2, neuronal | 363.9 | Integral membrane protein |
|  | Superoxide dismutase 3, extracellular | 26.2 | Enzyme/antioxidant and helps in reducing ROS and delaying apoptosis [[23](#_ENREF_23)]. Influences the functional competence of cryopreserved spermatozoa [[24](#_ENREF_24)] |
|  | Follistatin-like 4 | 78.8 | Glycoprotein/minimizes apoptosis [[25](#_ENREF_25)] |
|  | Dihydropyrimidinase-like 2 | 58.1 | Enzyme |
|  | Pyruvate kinase, muscle | 56.9 | Enzyme |
|  | Phosphorylase, glycogen, liver | 97.4 | Enzyme |
|  | Actinin, alpha 1 | 102.4 | Actin binding protein/regulate cell survival by TNF-alpha-induced survival signalling [[17](#_ENREF_17)] |
|  | Spectrin, beta, non-erythrocytic 1 | 272.5 | Actin crosslinking and molecular scaffold protein |
|  | Spectrin, alpha, non-erythrocytic 1 | 283.7 | Actin crosslinking and molecular scaffold protein |
|  | Beaded filament structural protein 1, filensin isoform X2 | 74.2 | Intermediate filament |
|  | Beaded filament structural protein 1, filensin isoform X3 | 70.0 | Intermediate filament |
|  | Rho GDP dissociation inhibitor (GDI) beta | 45.4 | GDP dissociation inhibitor protein |
|  | Glutathione synthetase | 52.1 | Enzyme |
|  | Tissue specific transplantation antigen P35B | 36.0 | Enzyme |
|  | Ectonucleotide pyrophosphatase/phosphodiesterase 2 | 100.1 | Enzyme |
|  | Opticin | 35.7 | Small Leucine-Rich Repeat Protein |
|  | Complement factor H-related | 35.2 | Glycoprotein |
|  | Enolase 1, (alpha) | 47.3 | Enzyme |
|  | Calsyntenin 1 | 106.2 | Transmembrane protein |
|  | Agrin | 207.0 | Proteoglycan |
|  | Crystallin, beta B3 | 24.3 | Structural protein |
|  | Seizure related 6 homolog | 105.9 | Transmembrane receptor |
|  | WAP, Kazal, immunoglobulin, Kunitz and NTR domain-containing protein 2 isoform X2 | 53.0 | Globular protein |
|  | Aminopeptidase puromycin sensitive | 86.1 | Enzyme |
|  | Keratin 42, type I | 50.3 | Cytoskeletal protein |
|  | Ubiquitin-conjugating enzyme E2,O | 124.5 | Enzyme |
|  | Serpin peptidase inhibitor, clade A (alpha-1 antiproteinase, antitrypsin) | 46.1 | Enzyme/ inhibits apoptosis by regulating caspase-3 [[26](#_ENREF_26)] |
|  | Drebrin-like | 43.7 | Actin binding protein |
|  | Cell adhesion molecule L1-like | 130.3 | Transmembrane protein |
|  | Transketolase isoform X2 | 63.1 | Enzyme |
|  | Filamin B, beta isoform X5 | 275.3 | Globular protein |
|  | Filamin B, beta isoform X6 | 233.2 | Globular protein |
|  | Complement component 4A isoform X1 | 187.6 | Glycoprotein |
|  | Aldolase A, fructose-bisphosphate | 39.4 | Enzyme |
|  | N-terminal Xaa-Pro-Lys N-methyltransferase 1 | 69.7 | Enzyme |
|  | Cadherin-related family member 1 | 90.0 | Transmembrane protein |
|  | Amyloid beta (A4) precursor-like protein 2 | 80.9 | Serine-type endopeptidase inhibitor |
|  | Rho GDP dissociation inhibitor alpha | 45.5 | Inhibitor protein |
|  | Ubiquitin-like modifier activating enzyme 1 | 117.8 | Enzyme |
|  | Cytoplasmic protein | 13.6 |  |
|  | StbB stable plasmid inheritance protein B | 14.6 | Plasmid stability protein |
|  | Y box binding protein 3 | 48.5 | Transcription factor |
|  | Tubulin, beta 2B class IIb | 10.3 | Globular protein |
|  | ATPase, Na+/K+ transporting, alpha 1 polypeptide | 67.0 | Enzyme |
|  | LPS-assembly protein | 101.6 | Membrane assembly protein |
|  | Crystal structure of bovine serum albumin in complex with naproxen, chain B | 66.4 | Globular protein |

**References**

1. Shukla MK, Misra AK (2007) Effect of Bradykinin on Murrah buffalo (Bubalus bubalis) semen cryopreservation. Anim Reprod Sci 97: 175-179.

2. Gardino AK, Yaffe MB (2011) 14-3-3 proteins as signaling integration points for cell cycle control and apoptosis. Semin Cell Dev Biol 22: 688-695.

3. Rosenquist M (2003) 14-3-3 proteins in apoptosis. Braz J Med Biol Res 36: 403-408.

4. Rerole AL, Jego G, Garrido C (2011) Hsp70: anti-apoptotic and tumorigenic protein. Methods Mol Biol 787: 205-230.

5. Shifrin Y, Arora PD, Ohta Y, Calderwood DA, McCulloch CA (2009) The role of FilGAP-filamin A interactions in mechanoprotection. Mol Biol Cell 20: 1269-1279.

6. Kawaguchi T, Yamagishi S, Itou M, Okuda K, Sumie S, et al. (2010) Pigment epithelium-derived factor inhibits lysosomal degradation of Bcl-xL and apoptosis in HepG2 cells. Am J Pathol 176: 168-176.

7. Zhang H, Kim JK, Edwards CA, Xu Z, Taichman R, et al. (2005) Clusterin inhibits apoptosis by interacting with activated Bax. Nat Cell Biol 7: 909-915.

8. Eyer J, White D, Gagnon C (1990) Presence of a new microtubule cold-stabilizing factor in bull sperm dynein preparations. Biochem J 270: 821-824.

9. Fukuda A, Osawa T, Oda H, Tanaka T, Toyokuni S, et al. (1996) Oxidative stress response in iron-induced acute nephrotoxicity: enhanced expression of heat shock protein 90. Biochem Biophys Res Commun 219: 76-81.

10. Wang P, Wang YF, Wang H, Wang CW, Zan LS, et al. (2014) HSP90 expression correlation with the freezing resistance of bull sperm. Zygote 22: 239-245.

11. Germann A, Schulz JC, Kemp-Kamke B, Zimmermann H, von Briesen H (2011) Standardized Serum-Free Cryomedia Maintain Peripheral Blood Mononuclear Cell Viability, Recovery, and Antigen-Specific T-Cell Response Compared to Fetal Calf Serum-Based Medium. Biopreserv Biobank 9: 229-236.

12. Averill-Bates DA, Yée MC-S, Grondin M, Sarhan F, Ouellet F (2014) C-1003: Cryopreservation of rat hepatocytes with wheat proteins: Role in oxidative stress protection. Cryobiology 69: 512-513.

13. Burtnick LD, Urosev D, Irobi E, Narayan K, Robinson RC (2004) Structure of the N-terminal half of gelsolin bound to actin: roles in severing, apoptosis and FAF. EMBO J 23: 2713-2722.

14. Pages C, Simon MF, Valet P, Saulnier-Blache JS (2001) Lysophosphatidic acid synthesis and release. Prostaglandins Other Lipid Mediat 64: 1-10.

15. De Souza EM, Meuser-Batista M, Batista DG, Duarte BB, Araujo-Jorge TC, et al. (2008) Trypanosoma cruzi: alpha-2-macroglobulin regulates host cell apoptosis induced by the parasite infection in vitro. Exp Parasitol 118: 331-337.

16. Pinto VI, Senini VW, Wang Y, Kazembe MP, McCulloch CA (2014) Filamin A protects cells against force-induced apoptosis by stabilizing talin- and vinculin-containing cell adhesions. FASEB J 28: 453-463.

17. Triplett JW, Pavalko FM (2006) Disruption of alpha-actinin-integrin interactions at focal adhesions renders osteoblasts susceptible to apoptosis. Am J Physiol Cell Physiol 291: C909-921.

18. Hook VY, Hwang SR (2002) Novel secretory vesicle serpins, endopin 1 and endopin 2: endogenous protease inhibitors with distinct target protease specificities. Biol Chem 383: 1067-1074.

19. Ng KM, Lee YK, Lai WH, Chan YC, Fung ML, et al. (2011) Exogenous expression of human apoA-I enhances cardiac differentiation of pluripotent stem cells. PLoS One 6: e19787.

20. Han SW, Roman J (2006) Fibronectin induces cell proliferation and inhibits apoptosis in human bronchial epithelial cells: pro-oncogenic effects mediated by PI3-kinase and NF-kappa B. Oncogene 25: 4341-4349.

21. Glander HJ, Herrmann K, Haustein UF (1987) The equatorial fibronectin band (EFB) on human spermatozoa--a diagnostic help for male fertility? Andrologia 19: 456-459.

22. Vij N, Roberts L, Joyce S, Chakravarti S (2004) Lumican suppresses cell proliferation and aids Fas-Fas ligand mediated apoptosis: implications in the cornea. Exp Eye Res 78: 957-971.

23. Greenlund LJ, Deckwerth TL, Johnson EM, Jr. (1995) Superoxide dismutase delays neuronal apoptosis: a role for reactive oxygen species in programmed neuronal death. Neuron 14: 303-315.

24. Waheed MM, Gouda EM, Khalifa TA (2013) Impact of seminal plasma superoxide dismutase and glutathione peroxidase on cryopreserved buffalo spermatozoa. Anim Reprod Sci 142: 126-130.

25. Liang X, Hu Q, Li B, McBride D, Bian H, et al. (2014) Follistatin-like 1 attenuates apoptosis via disco-interacting protein 2 homolog A/Akt pathway after middle cerebral artery occlusion in rats. Stroke 45: 3048-3054.

26. Petrache I, Fijalkowska I, Medler TR, Skirball J, Cruz P, et al. (2006) alpha-1 antitrypsin inhibits caspase-3 activity, preventing lung endothelial cell apoptosis. Am J Pathol 169: 1155-1166.
